# Nurse managers' perceptions about supportive work environment in public hospitals in Saudi Arabia: a cross-sectional study

**DOI:** 10.3389/fpsyg.2025.1569486

**Published:** 2025-06-10

**Authors:** Ahlam A. Almabadi, Olfat A. Salem, Ahmad E. Aboshaiqah, Naif H. Alanazi

**Affiliations:** ^1^College of Nursing, King Saud University, Riyadh, Saudi Arabia; ^2^Public Health Nursing Department, Faculty of Nursing, King Abdulaziz University, Jeddah, Saudi Arabia; ^3^College of Applied Medical Sciences, King Saudi University, Riyadh, Saudi Arabia

**Keywords:** nurse managers, perception, work environment, Saudi Arabia, support

## Abstract

**Background:**

An optimal work environment is essential for the effective performance of healthcare workers, particularly nurse managers, in order to achieve high-quality services. The current study aims to assess and compare nurse managers' perceptions of the adequacy of support within their work environment and availability of resources in four main hospitals in the Kingdom of Saudi Arabia.

**Subjects and methods:**

Through a cross-sectional study design, 260 nurse managers from four main general hospitals in Jeddah and Riyadh were selected using convenience sampling and invited to participate in the study. Their perception of the work environment was assessed using the Quality Work Environment Study (QWEST) questionnaire, which has been proven valid and reliable. Data were stored and analyzed using the Statistical Package for the Social Sciences (SPSS, Version 26) software. In addition to descriptive statistics, independent sample *t*-tests and ANOVA were used to test the significance of the differences between subgroups. Statistical significance was set at a *P* value of < 0.05.

**Results:**

The perception of nurse managers toward items reflecting work environment ranged from moderate (mean = 3.72, SD = 0.678) regarding support from supervisors regarding acting on values, even if it was at personal cost, to too high to support teamwork to achieve goals and outcomes (mean = 4.13, SD = 0.621). There was no statistically significant difference in the perceived work environment in the studied hospitals. Non-Saudis had a higher positive perception of the work environment (mean = 4.0, SD = 0.52) than Saudis (mean = 3.8, SD = 0.50), *P* = 0.006. They also showed moderate perceptions of the availability of resources and logistics, with no statistically significant differences between hospitals except for the availability of clerical support staff.

**Conclusion:**

Nurse managers generally perceive a moderate-to-high level of empowerment and support, indicating a positive environment across general hospitals. Non-Saudi nurse managers viewed their work environments more positively than Saudi nurse managers did. Although all hospitals had uniform resources, there was variance in clerical support between them. Hospitals should empower clerical staffing levels and adjust them to reduce administrative burdens to enable nurse managers to focus more on clinical leadership and patient care.

## 1 Introduction

The work environment in the nursing profession is defined as a system that supports nurses in controlling the delivery of nursing care, care setting, and organizational characteristics that either facilitate or hinder competent nursing practice (Lake, 2002). In recent years, Montgomery and Patrician identified three key elements that characterize a positive work environment: a “culture of generativity,” “adequate budgeted resources,” and a fair and manageable workload (Montgomery and Patrician, 2022). A well-structured work environment can help mitigate stress and promote resilience, which is defined as the ability to adapt to and recover from unexpected challenges (Al Sabei et al., 2021). Resilience and supportive environments are crucial in protecting nurse managers from burnout. Conversely, a poor work environment can negatively impact job satisfaction and effectiveness and increase turnover intentions (Montgomery and Patrician, 2022). A positive work environment not only improves patient care quality but also enhances workforce engagement and retention (Miller, 2020).

A positive work environment is multidimensional and characterized by trust, cooperation, safety, risk-taking support, accountability, and equity (Bryant, 2015). It encompasses elements such as empowerment, supervisory support, teamwork, feedback, stress management, mentorship, and autonomy in decision-making. Empowerment, in this context, refers to the ability to make informed decisions, access necessary resources, and receive opportunities for career growth (Lindberg and Vingård, 2012). In this respect, nurse managers play a vital role in creating and maintaining a positive work environment that ensures consistent delivery of safe, high-quality care. Their responsibilities include mentoring and coaching junior nurses to promote professional growth, as well as mediating staff conflicts to sustain a peaceful work environment (Alharbi, 2022; Duffield et al., 2015; Mota et al., 2021). Nurse managers hold various titles depending on their organizational roles, such as middle managers, patient services managers, clinical managers, or front-line managers (Miri et al., 2014). Mota et al. outlined the key leadership roles of nurse managers, which include acting as mentors, directors, coordinators, producers, monitors, brokers, facilitators, and innovators (Mota et al., 2021).

In today's complex healthcare settings, nurse managers are often selected on the basis of their clinical expertise. However, they may lack the formal training necessary for leadership roles, which require a broad skill set. Engaging in professional development activities that focus on advanced leadership skills can enhance their effectiveness in critical positions (Miltner et al., 2015). Although nurse managers play a partial role in shaping the work environment, they are affected by the overall work environment. For instance, Keith et al. found that insufficient work environment support, particularly inadequate administrative backing, was a major factor affecting nurse managers' job satisfaction (Keith et al., 2021). Furthermore, the absence of orientation programs, lack of knowledge, and insufficient skill development are associated with nurse managers' intentions to leave their roles (Keith et al., 2021). Moreover, according to the social exchange theory, the quality of work relationships depends on the mutual provision and receipt of resources and support in nursing management. This theory implies that when nurse managers are supported and recognized by their organizations, they are more likely to be committed and provide supportive leadership in return (Tran et al., 2018).

While previous research has emphasized the significance of a positive work environment for nurse managers, most attention has been focused on broad healthcare facilities, especially in Western countries. Nevertheless, research examining the distinct perceptions of nurse managers in Saudi Arabian public hospitals regarding the sufficiency of support within their work environment is lacking. Variations in culture, organization, and healthcare systems may have a substantial impact on the elements that influence these perspectives. Therefore, this study aims to assess and compare the perceived work environment support in public hospitals from the perspective of nurse managers in the Kingdom of Saudi Arabia (KSA).

## 2 Materials and methods

This study employed a cross-sectional, exploratory, and descriptive correlational design. The research was conducted in four major hospitals: two hospitals from Jeddah, denoted as H1 and H2, and two hospitals from Riyadh, denoted as H3 and H4. Riyadh is the capital, and Jeddah is the main seaport of the KSA. These hospitals were chosen because they have a high number of employees, including both Saudi and non-Saudi nurses, and large bed capacity. The minimum sample size needed (*N* = 103) was calculated using G^*^Power with a significance level of 0.05, 0.80, and a medium effect size of 0.15. A convenient method was used to select samples from the designated hospitals. The inclusion criteria were charge nurses, head nurses, nurse supervisors, area managers, and nursing directors present during the data collection. The exclusion criteria were nurse managers of less than a year in their units who lacked English literacy.

The quota sampling technique was used to determine the number of participants to be included from each department/unit in each building in the hospital. The quota sampling method was selected to obtain the maximum benefit, which mainly involved a large number of subjects and was more efficient in time. However, to minimize the negative aspect of the expected error, the inclusion criteria were decided based on the requirements for fulfilling the study aim. To determine the study sample size, an expert statistician was consulted.

The Quality Work Environment Study (QWEST) Questionnaire for nurse managers was used; it was developed using the Resonant Leadership Scale (Cummings et al., 2008). It included questions about work unit information, unit staffing, and resources. Furthermore, demographic factors of the nurse manager comprised age, gender, educational attainment, and professional history. Hewko et al. (2015) reported that the scale demonstrated internal consistency, with a Cronbach's alpha value of 0.66. Managers were required to express their level of agreement on a 5-point scale that included options from “strongly disagree” to “strongly agree.” Data were stored and analyzed using SPSS (Version 26) software for data management purposes. Scores were determined for each individual item, as well as for the overall level of agreement, with a rating of 5 indicating strong agreement and a rating of 1 indicating strong disagreement. The nurse managers' level of agreement on having a supportive work environment in their hospitals was tested for significant differences using independent-sample *t*-tests and ANOVA. Statistical significance was set at a *P* value of < 0.05. Approval was granted by the study settings to conduct the research.

Official written approval to conduct the study was obtained from the administration and research/ethical committee in selected settings (IRB Nos.: KSU-HE-23-769, KACST H-01-R-053, and NCBE: HA-02-J-008), as well as administrative personnel at the target hospitals. The official permission from the authors of the tools was also obtained to use the questionnaire to collect data for the current study. The validity of the tool was then established and conducted in order to assure the reliability and consistency of the questionnaire. The validity assessment focused on the visual characteristics of the QWEST instrument, such as formatting, readability, stylistic consistency, and clarity of terminology for nurse managers. Twenty-one nurse managers from hospitals that were not involved in the main study were invited to participate in the pilot study. They were asked the following question: “Are the questions and terminology clear?” If your answer is “No,” please specify which questions or terms were unclear. This inquiry was strategically positioned at the end of each page, allowing nurse managers to provide feedback before advancing to the next section of the questionnaire. Upon completing the survey, the nurse managers were asked three additional questions: (1) “Is the questionnaire user-friendly and easy to navigate?” to which all 21 nurse managers responded positively; (2) “How long did it take you to complete the survey?” with completion times reported between 20 and 30 min (average time = 25 min); and (3) “Do you have any further comments?” to which no additional feedback was provided by the 21 nurse managers who participated in the pilot study. The reliability assessment demonstrated satisfactory outcomes, with Cronbach's alpha values ranging from 0.88 to 0.95 for individual items of the questionnaire. Immediately after the pilot study and revision, the self-administered questionnaire was distributed to subjects who met the inclusion criteria during their on-duty shifts in the selected settings. Data collection took 8 weeks, from 10/1/2024 to 10/3/2024. A total of 324 questionnaires were sent to the hospital and manually distributed by the researcher, resulting in the return of 260 questionnaires, which corresponds to a response rate of 80.25%.

The ethical codes of conduct were strictly adhered to at all stages of the study. Regarding ethical issues pertaining to participants' consent to participate, having sufficient information regarding the research, and having the power to withdraw from the study at any stage, a written cover letter verifying the purpose of the study and the type of data that would be collected and ensuring anonymity and confidentiality of the subject was attached to each questionnaire. Informed consent that confirmed the participants' understanding of the information in the cover letter and signing the consent form was considered acceptable participation. Participants were informed that participation was voluntary and that they had the right not to answer any question(s) or withdraw from the study at any stage without any penalty. There were no apparent risks or benefits to the participants in this study. The researcher maintained the anonymity of the participants by removing all names and identifying information.

## 3 Results

Table 1 presents the mean and standard deviation (SD) of the nurse managers' responses regarding the support they received in their work environments. Overall, the results suggest that nurse managers feel relatively empowered and supported in their roles, with specific strengths and areas for potential improvement highlighted by individual items. Nurse managers moderately agreed that their work environment empowered them to accomplish their tasks effectively (mean = 3.86, SD = 0.695). A similar score (mean = 3.88, SD = 0.723) for perception of the workplace as empowered was consistent. Nurse managers slightly agreed (mean = 3.85, SD = 0.771) that their supervisors sought feedback. Many supervisors are value-driven; some nurse managers feel that personal costs affect their supervisors' ability to always act on values (mean = 3.72, SD = 0.761). The higher score for focusing on successes (mean = 4.01, SD = 0.678) reflects a positive perception that supervisors emphasize successes rather than failures, which is crucial for maintaining morale and motivation. The high score for support for teamwork (mean = 4.13, SD = 0.621) suggests that supervisors are perceived as strong advocates of teamwork. Regarding handling stress and communication, calmly handling stress (mean = 3.99, SD = 0.669) indicates that nurse managers generally believe that their supervisors manage stress well, contributing to a stable work environment. In addition, the mean score for active listening and acting on concerns (mean = 4.03, SD = 0.642) is seen as that of an effective listener who acts on requests and concerns, indicating strong communication skills and responsiveness. The high mean score of mentorship and decision-making (mean = 4.01, SD = 0.631) indicates that nurse managers feel that they receive active mentorship and coaching, suggesting a well-established support system for professional growth. Meanwhile, the mean value of conflict resolution (mean = 4.03, SD = 0.666) means that supervisors are seen as effective in resolving conflicts, which is critical for maintaining a harmonious work environment. In addition, the mean value of shared vision (mean = 4.01, SD = 0.652) indicates that engaging in a shared vision is positively rated, indicating alignment between supervisors and nurse managers in goals and direction. The freedom in decision-making (mean = 3.96, SD = 0.688) reflects that, while nurse managers feel relatively empowered to make decisions, the score suggests that there might be restrictions that could be improved to foster greater autonomy. Nurse managers believed that their orientation when starting their roles was adequate (mean = 3.87; SD = 0.753). Finally, an equal response was observed in mentorship from senior colleagues (mean = 3.87, SD = 0.768).

**Table 1:** Responses of nurse managers to items describing the supporting work environment.

**Items**	**Mean**	**SD**
Overall, my current work environment empowers me to accomplish my work in an effective manner	3.86	0.695
Overall, I consider my workplace to be an empowering environment	3.88	0.723
The supervisor/director that I report to…—Looks for feedback on ideas and initiatives even when it is difficult to hear	3.85	0.771
The supervisor/director that I report to…—Acts on values even if it is at a personal cost	3.72	0.761
The supervisor/director that I report to…—Focuses on successes and potential rather than failures	4.01	0.678
The supervisor/director that I report to…—Supports teamwork to achieve goals and outcomes	4.13	0.621
The supervisor/director that I report to…—Calmly handles stressful situations	3.99	0.669
The supervisor/director that I report to…—Actively listens, acknowledges, and then acts on requests and concerns	4.03	0.642
Actively mentors and coaches individual and team performance	4.01	0.631
The person you primarily report to at work—Effectively resolves conflicts that arise	4.03	0.666
Engages me in working toward a shared vision	4.01	0.652
Allows me freedom to make important decisions in my work	3.96	0.688
When I became a manager, my orientation was adequate	3.87	0.753
I receive mentorship from my senior colleagues in this managerial role	3.87	0.768

Table 2 shows the mean and standard deviation (SD) of the perceived supportive work environment of nurse managers across various characteristics. There was a higher mean score among males (mean = 4.1, SD = 0.60) than among females (mean = 3.9, SD = 0.50); however, this difference was not statistically significant. Non-Saudi nurse managers reported a significantly more positive perception of their work environment (mean = 4.0, SD = 0.52) than Saudi nurses (mean = 3.8, SD = 0.50), with a *P* value of 0.006. Regarding age categories, the mean scores range from 3.8 to 4.1, *P* = 0.539; however, there was no statistically significant difference across age groups (*P* = 0.539). In addition, there was no statistically significant difference in perceptions of a supportive work environment based on education level (*P* = 0.290). However, managers with postgraduate education reported slightly lower satisfaction (mean = 3.8) than those with a diploma or a bachelor's degree. Regarding the hospital, the mean scores range from 3.9 to 4.1, *P* = 0.089. Although there is no significant difference across hospitals (*P* = 0.089), H4 has a slightly higher mean (4.1) compared to the other hospitals. Moreover, according to the department, the mean scores range from 3.8 to 4.0, *P* = 0.478; however, there was no statistically significant difference across departments (*P* = 0.478). Departments such as medical and ICU have higher perceived support (mean = 4.0), while CCU and outpatient report slightly lower means (3.8).

**Table 2:** Differences in perceived supporting work environment according to hospital and characteristics of the nurse managers.

**Characteristics**	**Supportive work environment**		***P* value**
	**Mean**	**SD**	
**Gender**			
Male	4.1	0.60	0.153
Female	3.9	0.50	
**Nationality**			
Saudi	3.8	0.50	0.006^*^
Non-Saudi	4.0	0.52	
**Age categories**			
< 30 years	3.8	0.51	0.539
30– < 35 years	3.9	0.51	
35– < 40 years	4.0	0.47	
40– < 45 years	3.9	0.52	
45– < 50 years	4.1	0.55	
≥50 years	3.9	0.61	
**Education level**			
Diploma	4.0	0.46	0.290
Bachelor	4.0	0.51	
Postgraduate	3.8	0.59	
**Hospital**			
H1	3.9	0.48	0.089
H2	3.9	0.44	
H3	3.9	0.51	
H4	4.1	0.58	
**Department**			
Medical	4.0	0.49	0.478
Surgical	3.9	0.61	
ICU	4.0	0.43	
CCU	3.8	0.48	
NICU	4.0	0.87	
Outpatient	3.8	0.48	
Others	3.9	0.47	

^*^Statistically significant.

Table 3 presents the mean and standard deviation (SD) of nurse managers' perceptions of the availability of logistics and resources across the four hospitals. Regarding resources, the mean scores ranged from 2.8 to 3.0, *P* = 0.114, with no statistically significant difference (*P* = 0.114), which indicates that the availability of resources is generally perceived as similar across all hospitals. Regarding space, the mean scores ranged from 2.7 to 3.0, *P* = 0.226. Although H3 had a slightly higher mean (3.0), the overall perception suggests that space is moderately available, with no significant difference (*P* = 0.226). The mean score for the availability of clinical staff ranged from 2.9 to 3.0, *P* = 0.867, and H3 had a slightly higher score (3.0); the difference was not statistically significant. In contrast, regarding the availability of clerical staff, the mean scores range from 1.8 to 2.4, *P* = 0.017. This was the only statistically significant difference (*P* = 0.017). H1 had the lowest mean (1.8), whereas H3 had the highest (2.4). This variation suggests that clerical staff availability differs significantly between hospitals. The mean score of availability of professional staff ranged from 2.8 to 3.2, *P* = 0.159, which was rated moderately across hospitals, with H1 and H3 scoring higher (3.2). However, this difference was not statistically significant (*P* = 0.159). The educational support mean scores ranged from 2.8 to 3.5, *P* = 0.281; it was rated the highest in King Saud Medical City (3.5), although the difference between hospitals was not statistically significant (*P* = 0.281). However, H2 scores were lower (2.8), indicating a potential gap in support.

**Table 3:** Differences in perceived availability of logistics and resources according to different hospitals.

**Logistics**	**Hospital**				
	**H1**	**H2**	**H3**	**H4**	***P*** **value**
	**Mean** ±**SD**	**Mean** ±**SD**	**Mean** ±**SD**	**Mean** ±**SD**	
Resources	2.8 ± 0.46	2.8 ± 0.79	3.0 ± 0.77	2.8 ± 0.67	0.114
Space	2.8 ± 0.89	2.7 ± 0.98	3.0 ± 0.90	2.8 ± 0.94	0.226
Clinical staff	2.9 ± 0.77	2.9 ± 0.91	3.0 ± 0.80	2.9 ± 0.90	0.867
Clerical staff	1.8 ± 1.02	2.3 ± 1.23	2.4 ± 1.07	2.1 ± 1.20	0.017^*^
Professional staff	3.2 ± 0.65	2.8 ± 1.19	3.2 ± 0.97	3.1 ± 0.91	0.159
Educational support	3.3 ± 0.69	2.8 ± 1.03	3.5 ± 1.01	3.2 ± 0.87	0.281

^*^Statistically significant.

## 4 Discussion

Understanding the root factors of workplace challenges is essential for improving employee satisfaction and increasing their intention to stay, particularly among nurse managers. Identifying and addressing challenges such as workload distribution, communication barriers, and resource limitations allows nurse managers to establish a work environment that is more supportive and conducive. The current study aimed to assess and compare the perceptions of nurse managers about a supportive work environment in four main general hospitals in the KSA using the QWEST Questionnaire, which has been proven to be valid and reliable.

The results revealed an average moderate level of agreement among nurse managers about empowerment and support in their roles, which indicates that, although nurse managers feel somewhat empowered, there is still potential for enhancement. Nurse managers generally feel their work environment allows them to effectively complete tasks, despite some variability, possibly because of various experiences with resources, administrative support, or workload management in their specific work settings (Al Sabei et al., 2021; Alotaibi AGA toom, 2022; Alsufyani et al., 2021).

The substantial agreement of nurse managers that their supervisors prioritize success over failure is an essential element in sustaining motivation and morale. Highlighting achievements contributes to creating a favorable work atmosphere, promoting a feeling of fulfillment among staff members, leading to increased involvement and efficiency levels. Recent studies have shown that employees are more likely to improve overall organizational performance when they feel valued and appreciated for their accomplishments (Mazzetti and Schaufeli, 2022). In addition, supervisors' capacity to manage stress is important to uphold a steady work environment. This skill helps in decreasing tension among teams, fostering a culture of psychological safety, and preventing stress from affecting team members. Emphasizing mental health and wellbeing, a common trend in healthcare organizations, highlights the crucial leadership qualities needed for ongoing employee satisfaction and retention (Hallam et al., 2023).

Teamwork support is equally important in healthcare settings, as was evident from the higher scores for promoting teamwork. Teams that believe that their supervisors are supportive work together more cooperatively and efficiently. This empowered work environment that emphasizes mutual support, which is the typical relationship between healthcare professionals, leads to more effective health (Babiker et al., 2014).

The results showed a high mean score for active listening and acting on concerns. This ability is important for cultivating a favorable and attentive work environment. Practicing active listening enables leaders to establish a trustworthy and transparent environment, which leads to increased employee work engagement. Furthermore, utilizing efficient listening techniques, such as collecting immediate feedback and taking action, allows leaders to tackle issues before they worsen, leading to a workforce that is more motivated. These actions help create a dynamic and flexible corporate culture necessary for achieving high-quality health care (Mabona et al., 2022).

Non-Saudi nurse managers generally perceived their work environment as more positive than their Saudi colleagues. This indicates that nurse managers from non-Saudi backgrounds may feel more supported, independent, or have a better work environment than local nurse managers, possibly because of differences in expectations, cultural adaptation, or different job responsibilities in their workplaces. This difference can be attributed to both professional and personal situations. Non-Saudi nurse managers frequently travel to Saudi Arabia under specific job agreements that offer significant financial security and a better quality of life than what individuals would likely have in their native countries (Almalki et al., 2012).

The perception of nurse managers regarding the availability of logistics and resources across hospitals showed non-significant variation among different hospitals, which suggests that these resources, such as supplies, equipment, and staff support, are generally consistent across hospitals. This indicates that resource allocation might be standardized or adequately balanced across different settings, leading to a uniform experience in terms of resource access for nursing staff. Such consistency is crucial for maintaining stable work environments and ensuring that care delivery is not hindered by resource disparities (Tran et al., 2018; Alotaibi AGA toom, 2022).

The significant differences, on the other hand, in nurse managers' opinions on the availability of clerical staff among hospitals suggest varying levels of institutional support. For example, H1 had lower average scores, indicating a lack of clerical support perception, whereas H3 had higher scores, suggesting better staffing levels. Variations in staffing levels can affect how work is distributed, as having more clerical staff on hand can lessen administrative tasks for nurse managers. This gives them more time to concentrate on clinical leadership and patient care (Kearney et al., 2016).

This study had certain limitations that should be considered. The generalizability of the results is somewhat limited due to the sampling method and its composition. Although this study included four public hospitals, it relied on a convenience sample of nurse managers who opted to participate in the survey. This self-selection may introduce bias, and the sample may not accurately represent all nurse managers in the KSA. Future research should aim for a more stratified sampling approach that ensures adequate representation. This study employed a cross-sectional design, capturing data at a single point in time. While this design is suitable for exploratory and descriptive objectives, as in the current study, it inherently restricts the ability to draw causal conclusions. There may be unmeasured confounding variables or bidirectional influences on the adequacy of support within the work environment of nurse managers. A longitudinal approach is necessary to examine how a supportive work environment develops over time and to identify causal relationships. In addition, reliance on self-reported survey data introduces inherent biases, as nurse managers may underreport or overreport their perceptions due to social desirability, recall inaccuracies, or personal interpretations of the questionnaire items. Future qualitative research is needed to address the potential inadequacies or biases in self-reporting, as nurse managers may withhold true perspectives when answering questionnaires. This study specifically targeted nurse managers, which is a strength in terms of focusing on a particular work environment. However, this limits the findings' applicability to a broader healthcare workforce. The results may not apply to nurses in positions higher than nurse managers or to other hospital settings in different regions of the KSA, including private hospitals. Consequently, caution should be exercised when inferring the applicability of these findings beyond public hospital contexts and at the national level. Finally, a more extensive survey that includes all nurses in managerial and higher positions, those in private hospitals, and those across the country is recommended.

## 5 Conclusions and recommendations

Nurse managers have a moderate consensus on empowerment and support, indicating the potential for enhancement of their responsibilities. Although nurse managers had different experiences with resources and administrative support, they typically found their work environments supportive of task completion. The study highlighted the importance of supervisors focusing on accomplishments to uphold motivation and a positive work environment. Supervisors' skills in handling stress help create a stable environment and foster a culture of psychological safety in healthcare leadership, prioritizing mental wellbeing. Teamwork was also emphasized, showing high ratings for the positive effects of working together. Being attentive and responsive to staff concerns is crucial for fostering trust and transparency, which ultimately boosts engagement. Non-Saudis showed a more positive perception of the work environment. Uniform resource availability in hospitals leads to consistent work environments; however, differences in clerical support levels impact nurse managers' allocation of time between clinical and administrative tasks.

Based on the variations in perceived clerical support that could impact nurse managers' workload, hospitals should assess staffing levels and adjust them to reduce administrative burdens. This would enable nurse managers to focus more on clinical leadership and patient care. Further research into why non-Saudi nurse managers perceive their work environment more positively can inform strategies to improve perceptions among Saudi nurse managers.

## Data Availability

The original contributions presented in the study are included in the article/supplementary material, further inquiries can be directed to the corresponding author.
